# Addressing vulnerabilities in communities facing infectious disease threats: A need for social science-driven assessments

**DOI:** 10.7189/jogh.11.03003

**Published:** 2021-01-13

**Authors:** Jacob Osborne, John Paget, David Napier, Tamara Giles-Vernick, Ruth Kutalek, Roman Rodyna, Syed Masud Ahmed, Michel Dückers

**Affiliations:** 1 Nivel – Netherlands Institute for Health Services Research, Utrecht, the Netherlands; 2 Department of Anthropology, Science, Medicine, and Society Network, University College London, London, UK; 3 Anthropology and Ecology of Disease Emergence Unit, Institut Pasteur, 75015 Paris, France; 4 Department of Social and Preventative Medicine, Center for Public Health, Medical University of Vienna, Vienna, Austria; 5 Public Health Center of the Ministry of Health of Ukraine, Kyiv, Ukraine; 6 James P. Grant School of Public Health, BRAC University, Dhaka, Bangladesh; 7 ARQ National Psychotrauma Centre, Diemen, the Netherlands; 8 Faculty of Social and Behavioural Science, University of Groningen, Groningen, the Netherlands

In the current COVID-19 crisis, global and national public health authorities and organisations are searching their toolbox of methods and approaches to communicate to and connect with populations. As with HIV/AIDS or Ebola Virus Disease (EVD) in West Africa, it became quickly apparent that the participation of those most affected by the disease, and communities in general, play a central role in understanding how to best shape and implement response efforts [1,2]. The same holds true for COVID-19 in 2020: there is an urgent need to understand the ways public health measures and interventions reach and affect communities and how these heterogenous and complex groups can contribute to an effective response in times of crisis. This recognition of the importance of engaging with communities has proved to be the point where the social sciences may again play a crucial role.

Biomedical and epidemiological research on infectious diseases currently informs much of the surveillance and response efforts in disease outbreaks and is readily taken up as evidence for implementing health programming across the globe. Social science scholarship, on the other hand, is historically a more marginalised discipline within epidemic responses but has an important role to play, ranging from anthropological assessments, economic analyses, policy recommendations, and communication strategies [3]. Moreover, the use of social science to assess community engagement in epidemics brings to light how individuals constitute or are left out of local communities, further necessitating an understanding of vulnerabilities to outside threats. Social science can provide not only appropriate methods for working with communities, but also the theoretical and experiential knowledge that adds to a fruitful and empowering engagement process.

The European Commission is one among other international bodies that have recognised this opportunity by funding the consortium Sonar-Global, a Global Social Sciences Network for Infectious Threats and Antimicrobial Resistance (AMR), to form a network for preparedness and response to epidemics and AMR, providing governance, tested tools and capacity strengthening efforts for infectious threats [4]. Likewise, the objective of this commentary is to: 1) highlight the need for community engagement that recognises vulnerability, and 2) subsequently encourage the use of social science as a way to increase the impact of public health responses to infectious disease outbreaks.

## AN INTEGRATED FRAMEWORK FOR INFECTIOUS DISEASE EPIDEMICS

The integration of the fields of traditional infectious diseases and social science provide a promising approach for the productive utilisation of community engagement as it relates to the mitigation of particularly situated vulnerabilities, including unequal access to vital resources. We examine here four elements surrounding these issues within epidemic responses and propose an integrated framework that can inform current and future responses to infectious disease epidemics. The four elements include: traditional infectious disease science, social science, community engagement, and vulnerability.

### 1. Traditional infectious disease science

An infectious disease that causes the epidemic or outbreak is the content or subject of what the other elements of this integrated approach can speak to. Research related to infectious disease is dominated by the biomedical, epidemiological, and public health fields, which grant us the tools to discover the natural history, transmission, diagnosis, and treatment of infectious diseases. These disciplines, however, are not necessarily uniform in their methods and messages, which has become clear given the mixed and sometimes contradicting advice from researchers and public health authorities during the COVID-19 pandemic. Other non-clinical fields can aid in forming a response that prioritises multi-sectoral health in all policies and universal health coverage.

### 2. Social science

Social science, including such disciplines as anthropology, sociology, history, and science and technology studies, interrogate social processes in particular contexts, filling a gap left by traditional infectious disease science. Social sciences within HIV research, for example, enjoys a relatively rich body of scholarship and practice, especially in terms of engaging with certain groups and in communicating risk around HIV transmission [5]. Anthropology in epidemics can also offer, as Stellmach et al. (6, p. 3) propose, “insight on why public health interventions succeed or fail: the gap between what is planned and what is realised on the ground and the unintended consequences that may result. [6]” Indeed, it is through ethnographic methods that anthropologists can add depth to understandings of communities located within particular social, political, economic, and institutional worlds. The processes through which communities navigate these worlds are necessarily entangled in how they encounter the challenges of a new epidemic or infectious threat.

**Figure:**
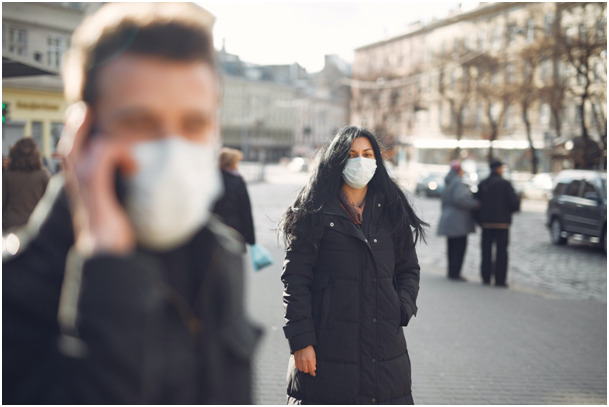
Photo: https://www.pexels.com/photo/woman-in-black-coat-and-face-standing-on-street-3983428/.

### 3. Community engagement

Community engagement as a methodological framing is not a new concept – it has been invoked in many forms in many contexts [7] and is an emerging tool being used in epidemic responses. Community engagement is recognised as an important part of responding to epidemics in a way that is culturally sensitive and protects the safety of both those affected by disease it (eg, HIV prevention messages for men who have sex with men) as well as health care workers. It has earned the attention of international organisations that implement it within programs aimed at empowering social networks and strengthening local capacities, although at varying levels of engagement or even with varying conceptions of what is meant by “community” or “engagement” [8]. Here, it is clear that social science constructions of these concepts are useful, if not necessary for the method’s coherency and successful implementation, especially considering that those most vulnerable often do not have the capacity to engage in behaviour change interventions.

### 4. Vulnerability

Vulnerability is a concept that offers an important connection between the three previously described elements. One can be vulnerable to acquiring an infectious disease due to certain biological or demographic factors, but this vulnerability can also be considered at the social or structural levels – domains of social science that also involve investigating social capital or access to health services, for instance [9]. Located at the intersection of the social dimensions of resilience to infectious disease and epidemiologically described risk, vulnerability is a strategic concept that must be addressed within community-based interventions. It is thus closely related to (and even reflects a lack of) community engagement and relies on gaining a nuanced and realistic assessment of how those who are most vulnerable are affected by epidemics. Some very useful methods grounded in social science theory have been developed to assess vulnerability locally [10].

**Figure 1:**
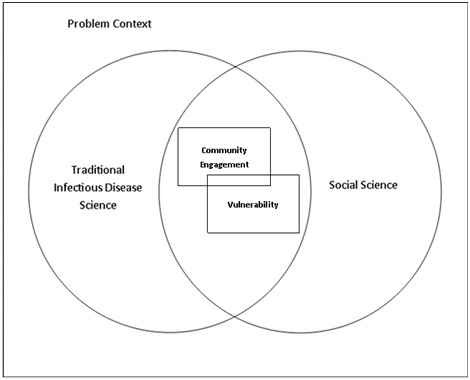
An integrated framework for research and action in infectious disease threats. Within the context of epidemics or outbreaks, two lenses (traditional infectious disease science and social science) and two concepts (community engagement and vulnerability) are closely interconnected. This figure represents an approach that emphasises the added value of social science and the importance of recognising vulnerability within community engagement efforts. Ideally, a response or risk mitigation approach should address both concepts through both lenses.

Separately, the four elements described above offer broad bodies of knowledge in terms of literature, protocols, and methods. Clearly, within the context of epidemics or outbreaks, the four are closely interconnected and responses should reflect and integration of each element. **Figure 1** illustrates the four elements where two fields, traditional infectious disease science and social science, intersect along the lines of vulnerability and community engagement, which can be considered within a particular context (eg, COVID-19, EVD in West Africa, etc.). The practical implications of such a framing suggest an integrated and effective approach that uses community engagement informed by vulnerability, grounded in knowledge from both social science and traditional infectious disease science.

## IMPLICATIONS FOR RESEARCH, POLICY AND PRACTICE

It should be noted that although community engagement and vulnerability are illustrated here as two distinct concepts, importantly, they are closely interrelated and one should inform the other in practice. For example, responses to the COVID-19 pandemic may assess constraints and vulnerabilities experienced by certain groups and engage with communities to shape appropriate and effective prevention measures that help mitigate vulnerabilities. Blanket measures such as containment, isolation and quarantine may also stoke fear and unintended reactions. Further, this framing is not meant to suggest a fixed relationship between the four elements or characterise their scope, but rather present a practical framework that can provide some coherency and guidance to epidemic responses. Government and health authorities, health care organisations, and researchers can use this focus to contribute to a robust and more complete response to infectious diseases threats: from preparation, to onset, to aftermath.

The integration of perspectives and research traditions necessitates a collaborative and multisectoral response, considering relational and structural aspects of infectious disease outbreaks. Whereas some community engagement efforts seek to reach specific groups and ensure that health interventions achieve changed attitudes or behaviours, we argue that the benefits of such behaviour change interventions do not necessarily address pertinent issues such as inequality and access to health care. The Sonar-Global consortium uses community engagement within infectious diseases outbreaks from a standpoint rooted in social science, recognising the particular ways that individuals and communities experience vulnerability and identifying relations and mechanisms that support community resilience to the challenges presented by infectious diseases. Such a context-specific, community-centred approach, however, has to date not been taken up as readily for the current COVID-19 epidemic.

## CONCLUSION

The framework presented above highlights the importance of using knowledge and methods from the social sciences to support prevention and control measures regarding infectious diseases. It has direct applications to epidemic responses as it provides a framing to map and plan activities in the context of infectious disease threats with special consideration for vulnerable groups and community engagement. Utilising such an integrated framing of the social science of vulnerability and community engagement may aid in achieving a more nuanced and inclusive approach to controlling infectious diseases. This framing stems from the motivation to connect currently separate pieces of infectious disease response and create a framework for research and action that is necessarily integrated, holistic, and community centred. In the case of Sonar-Global this includes the control of EVD in Central Africa, measles in Ukraine, and COVID-19 in Bangladesh. These are case studies to support the thought that the framework is also relevant to other global projects and initiatives designed to address the risk infectious diseases pose to all people across country borders.

Importantly, an approach that builds on community-focused measurements and the social dimensions of human vulnerabilities necessitates the serious consideration of social science concepts. This means that social scientists themselves should be part of the teams of NGOs, public health bodies and health professionals that make up the response to infectious disease epidemics. It is imperative that social science knowledge guides the assessment of vulnerability as a fuller and more effective response, which may complement biomedical and other traditional infectious disease fields. The integrated framework presented here therefore calls for a further reflection of what social science means in epidemics and puts forth a direction for thoughtful community engagement to more effectively control infectious diseases.
